# Energy Management for Personalized Weight Reduction (EMPOWER) Program: Three-Year Outcome Data

**Published:** 2019-03-10

**Authors:** AP Vidmar, C Fink, B Torres, B Manzanarez, SD Mittelman, CP Wee, C Borzutzky

**Affiliations:** 1 Center for Endocrinology, Diabetes and Metabolism, Department of Pediatrics, Children’s Hospital Los Angeles, Los Angeles, USA; 2 The Diabetes & Obesity Program, Children’s Hospital Los Angeles and The Saban Research Institute, Department of Pediatrics, Children’s Hospital Los Angeles, Los Angeles, USA; 3 Department of Pediatrics, David Geffen School of Medicine, University of California Los Angeles, Los Angeles, USA; 4 CTSI Biostatics Core, Saban Research Institute, Los Angeles, USA; 5 The Diabetes & Obesity Program, Division of Adolescent and Young Adult Medicine, Children’s Hospital Los Angeles and Keck School of Medicine of USC, Los Angeles, USA

**Keywords:** Obesity, Pediatrics, Weight Loss, Multi-Disciplinary Clinic

## Abstract

**Background:**

The current consensus guidelines for management of pediatric obesity recommend clinic-based, family-centered, multi-disciplinary interventions. It is well reported that these programs often only lead to modest improvements in BMI status. The individual factors that differentiate which patient’s BMI status will improve vs. worsen remains understudied. A retrospective cohort study was conducted to evaluate the outcomes of EMPOWER clinic and identify the participant specific characteristics that predicted BMI status improvement in this population.

**Methods:**

Youth who completed at least 6 visits in EMPOWER were included. Paired t-test was utilized to evaluate the mean change in zBMI, modified BMIz and %BMI_p95_ from baseline to 6^th^ visit, and multivariate mixed effect models were utilized to analyze effect of baseline characteristics on change in BMI status.

**Results:**

92 participants were included in the analysis, 87% with severe obesity and 66% Hispanic. At the 6^th^ visit, there was a significant reduction in zBMI (−0.09 SD, p <0.001) and modified BMIz (−0.0003 SD, p = 0.04) with a small reduction in %BMI_p95_ (−1.15 %, p = 0.20). Lower BMI status (p < 0.001) and absence of a comorbidity (p < 0.05) at baseline were predictors of BMI status improvement whereas age, gender, ethnicity, family history of obesity and insurance status were not significant predictors.

**Conclusions:**

Given that implementation of the current guidelines for management of obesity in pediatrics only results in modest BMI status reduction, further investigation is required to understand how the determinants of obesity-related health outcomes can guide development of more innovative, effective interventions for this high risk population.

## Introduction

Severe obesity (BMI ≥ 120% of the 95^th^ percentile) among children and adolescents is increasing [1]. Currently 3.8 to 5.4 million youth in the United States are severely obese [1–5]. Children with severe obesity are at increased risk of developing early-onset, life-limiting medical and psychological comorbidities and have higher rates of mortality [1,3,6,7]. This pediatric population is at high risk of poor health outcomes and therefore requires thoughtful study of the most effective treatment strategies to promote stabilization of their BMI trends. The recent consensus guideline for the treatment of pediatric obesity recommends comprehensive, multi-disciplinary, family- based interventions [8,9]. As described by both Janicke et al. and Zolotarjova et al., most of these comprehensive programs result in modest short-term improvement in BMI Z-score (zBMI) of −0.1 to 0.6 units from first to last visit (on average 6 visits, over a 6 month period of time) and have variable effect on maintenance of that improvement [9–12]. Not only is the degree of BMI status reduction often minimal but the intervention-mediated BMI changes can vary substantially between patients [13]. The cause of this variability remains understudied. Better characterization of the determinants of BMI status improvement after completion of a behavioral intervention could lead to improved effectiveness of clinical weight management programs and could facilitate their judicious use as adjuncts to the more definitive treatment options available such as pharmacotherapies and weight loss surgery.

There is a growing body of literature investigating both demographic and clinical predictors of treatment success in youth with obesity [14,15]. A recent systematic review by Zolotarjova et al. evaluated 16 studies conducted between 1995–2015, describing the effects of multidisciplinary weight management clinics on youth with severe obesity. As previously described, there appear to be several critical factors which predict treatment success including: age at start of treatment [14,16,17], gender [16,18,19], degree of obesity at baseline [20,21], and improvement in BMI within the first month of an intervention [11,22]. In separate reports by Knop et al., Danielsson et al. and Nowicka et al., study investigators showed that younger age at start of treatment and lower BMI status at baseline are positive predictors of BMI improvement over the course of treatment [14].

Interestingly, the findings from these studies are often contradictory: Reihner et al. reported that gender was not associated with change in zBMI at program completion, whereas Holm et al. reported that behavioral weight reduction programs were more successful in males [19,23]. These differing results may reflect the use of various BMI metrics to report effectives of the intervention or the variability of the outcomes utilized to measure program success [11,20,24]. Several studies have reported that zBMI may not be the best metric of intervention effectiveness for youth with severe obesity [25,26]. Freedman et al. and the CDC propose utilizing alternative metrics for weight change such as modified BMI z-score (modified BMIz) or %BMI_95_ which expresses BMI as a percentage of the 95^th^ percentile [25,27]. Review of the literature reveals that choice of metric (i.e. BMIz, modified BMIz, or %BMI 95), and to whom it is applied, may skew results. Additionally, regardless of BMI status metric utilized, assessment of other factors, such as cardiometabolic indicators (i.e. blood pressure, lipid and transaminase levels, and assessments for insulin resistance), quality of life, and physical activity may be required to fully evaluate the impact of these interventions on overall health status in youth with severe obesity [3,7].

In accordance with the 2007 American Academy of Pediatrics recommendation for pediatric weight management interventions, the Children’s Hospital Los Angeles (CHLA) Energy Management for Personalized Weight Reduction (EMPOWER) clinic was established in 2014 in response to the rising levels of childhood obesity in the Southern California region. EMPOWER is a multi-disciplinary family-centered weight management clinic comprised of physicians (general pediatricians, adolescent medicine obesity specialists, and pediatric endocrinologists), dietitians, psychologists, and physical therapists. The purpose of this study was twofold: 1) To evaluate change in zBMI, Modified BMIz and %BMI_p95_ from baseline to 6^th^ visit in youth participating in EMPOWER and 2) To evaluate how baseline demographics or clinical characteristics of treatment-seeking youth or their parents predicted treatment outcomes, across all three BMI status metrics, in this high risk population.

## Methods

### Study design

Study procedures were approved by the Children’s Hospital Los Angeles (CHLA) Institutional Review Board (CHLA-18–034). One parent or guardian of each subject provided written consent for their child’s information to be extracted from the medical record. All subjects gave assent to participate. A retrospective medical chart review was conducted of 532 unique patients enrolled in the CHLA EMPOWER Clinic. Eligible participants were seen at the EMPOWER clinic between January 2014 and December 2016.

### Empower clinic

The EMPOWER clinic is a multidisciplinary clinic designed for youth with obesity. Patients attend an initial visit where they are assessed by all four providers (MD, RD, PT, and Psychologist) and return for monthly follow-up visits. It is recommended that all patients attend a minimum of six visits. The visit length on average is 100 minutes at the initial visit and 80 minutes per follow up visit, providing approximately eight contact hours per participant per six-month intervention. Eligible patients are up to 21 years of age and meet one of the following criteria: 1) body mass index (BMI)-for-age ≥ 120% the 95^th^ percentile (severely obese), 2) BMI-for-age ≥ 95^th^ percentile (obese) with any obesity-related comorbidity, 3) BMI-for-age ≥ 85^th^ percentile (overweight) with significant obesity-related comorbidities requiring multidisciplinary care. Eligible comorbidities include the following: insulin resistance, hyperlipidemia/dyslipidemia, Non-Alcoholic Fatty Liver Disease (NAFLD), orthopedic problems (eg, Blount’s disease), Obstructive Sleep Apnea (OSA), hypertension (blood pressure > 99^th^ percentile for age and gender), Oligomenorrhea/Polycystic Ovary Syndrome (PCOS), and depression/anxiety.

Patients are evaluated on an individual basis by the clinic providers (MD, RD, PT, and psychologist). At the initial visit, each provider obtains a detailed history, and performs an assessment, focusing on his/her individual areas of expertise. Assessment may include past medical history, psychological/psychiatric history, as well as an inventory of parental history of obesity and Type 2 Diabetes (T2D), patient and family nutritional habits (including the number and timing of regular meals, details of snacks, energy-dense foods, soft drink consumption, and whether the child exhibits any binge-eating behaviors), physical activity (both formal and informal) sleep habits, coping mechanisms/stress management strategies, and sedentary activities (television watching/computer games). It also includes a review of systems, anthropomorphic measurements and vital signs, physical examination, fitness assessment by various metrics, and review of laboratory examinations. The team assesses for possible medical causes of the patient’s obesity, counsels regarding problems that may be anticipated, and recommends lifestyle changes that could lead to stabilization/improvement in BMI status. Appropriate medical referrals are made, including pulmonology and gastroenterology, for assessment of possible OSA and/or NAFLD. Changes in behavior for the entire family are strongly encouraged. This is in accordance with previous studies that have shown beneficial results to the child when the parents are involved [2].

The multi-disciplinary team utilizes treatment strategies that include motivational interviewing, nutritional education, and behavioral therapies which together target improving daily routines, emotional health, sleep quality and quantity, diet, and physical activity, and elicit how family members will support the patient in these changes [28]. Each team member documents the patient’s identified goals and a goal summary is provided to the team and the family at check-out. These goals are then followed up at subsequent monthly visits.

### Measures

#### Demographic data

Demographic information, such as patient age, sex, ethnicity, and insurance status, was abstracted from the patient Electronic Medical Record (EHR). For the insurance status variable, all individuals receiving government assistance were collapsed into the category Medi-Cal (equivalent of Medicaid in California).

#### Retrospective anthropometric data collection

Data collected from the electronic medical record included BMI and zBMI. %BMI_p95_ (a score of 100 is equivalent to the 95^th^ BMI percentile) was determined utilizing the CDC growth charts (SAS program, cdc-source-code.sas) [4,25,26]. Freedman et Al. examined differing weight metrics in youth with obesity and severe obesity and reported that %BMI_p95_ was superior to zBMI for evaluating the impact of obesity treatment in youth with severe obesity, because the change in zBMI of youth with severe obesity are weakly associated with change in body size [25,26]. In addition, the CDC recommends the use of modified BMI z-score in youth with severe obesity which is constructed by extrapolating one-half of the distance between 0 and 2 z-scores to more extreme values [4,5,25,26]. Therefore, all three metrics were evaluated in this study to further explore their use as outcome measures in youth with obesity and severe obesity.

### Clinical data

Clinical data including co-morbidities, family history, age at start of treatment, and blood pressure, were abstracted from the electronic medical record. Blood pressure values were categorized using national data from the Centers for Disease Control and the National Heart, Lung and Blood Institute Task Force on Blood Pressure Control in Children [9]. In addition, parental information was obtained, including history of one or more parent with: obesity, T2D, depression or anxiety.

### Outcomes

The primary outcome measures for this study were change in zBMI, modified BMIz, and %BMI_p95_ from baseline to 6^th^ visit. Secondary outcomes included correlation of weight outcome with the following covariates: age at start of treatment, gender, severity of obesity, obesity or nonobesity related co-morbidity, family history of obesity, T2D or depression and anxiety.

### Data analysis

**Figure 1:**
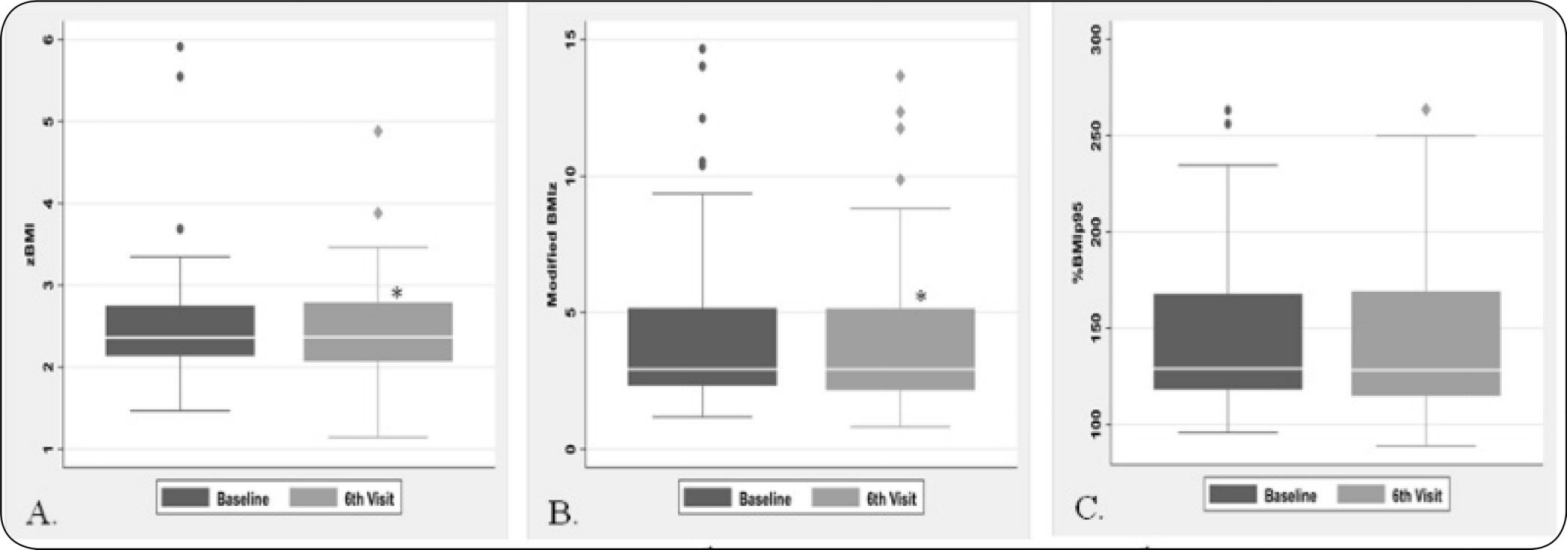
Change in BMI metric from baseline to 6^th^ visit. A. BMI Z-score at the 6^th^ visit decreased by-0.07 (0.25) SD (*p < 0.01). Data represents mean ± stdev changed compared to baseline. B. Modified BMI Z-score at the 6^th^ visit decreased by-0.0003 SD (CI = −0.72, 0.72,*p = 0.04). Data represents median ± stdev changed compared to baseline. C. %BMI_p95_ at the 6^th^ visit decreased by −1.15 (8.56) percentage points (p = 0.20). Data represent mean ± stdev changed compared to baseline.

Descriptive statistics were used to summarize the average and distribution of the variables. Normally distributed continuous variables were summarized as mean and standard deviation and non-normally distributed as median and inter-quartile range. Categorical variables were summarized as frequencies and percentages. Paired t-test was utilized to examine the change in zBMI, modified BMIz and %BMI_p95_ at baseline to 6^th^ visit which is shown in figure 1. In addition, the change from baseline to 6^th^ visit (zBMI, modified BMIz and %BMI_p95_) between co-variates was examined using analysis of variance (ANOVA) with repeated measures. To determine if there are demographic and clinical characteristics that predict clinical efficacy of the weight management intervention, multivariable mixed effect models were utilized. Statistical significance was set at 05 with two-sided test used throughout the analysis. (Stata Intercooled 13.1, College Station, Texas).

## Results

Between January 2014 and December 2016 there were 532 unique patient visits with 1759 clinic visits. 92 of the 248 patients (35%) who attended visit 1 completed the intervention, defined as having attended at least 6 monthly visits (the 6 visits were completed over a mean time of 7.8 months). On average, the program completers attended 9.1 visits total (range 6–17 visits). After completing 6 visits, the MD and family determine need for additional visits in the program based on BMI status progress and family motivation. The majority of the program completers were male (52% vs. 21%, p < 0.001), had more non-obesity-related co-morbidities at baseline (74.7% vs. 36.1%, p < 0.001) with lower rates of anxiety or depression in one or more parents (p=0.004) compared to non-completers. Eighty-seven percent of the program completers had severe obesity at baseline. Thirty-four percent of the program completers were Medi-Cal enrollees and 66% were Hispanic. The mean age was 12.11 years (SD: 3.64, range: 9.1–21.3).

The program completers reduced their zBMI from baseline to 6^th^ visit by −0.09 SD (p = 0.001), modified BMIz by −0.0003 SD (p = 0.04) and %BMI_p95_ by 1.56 (p = 0.17, Figure 1A and B). Intriguingly, unlike previous reports, reduction across all three BMI metrics was not associated with age at treatment initiation, ethnicity, or insurance status at baseline. Male participants showed a greater reduction in zBMI, Modified BMIz and %BMI_p95_ from baseline to 6^th^ visit compared to females (p < 0.001). Multivariate analysis revealed that youth with lower BMI status at baseline, when controlled for age, gender and co-morbidity showed a greater improvement in BMI status at the 6^th^ visit (p < 0.001).

### Comorbid conditions as moderators of weight outcome

Seventy-nine (85%) of the program completers had obesity related comorbidities at baseline, including NAFLD (38%), Hypertension (33%), OSA (11%), and T2D (2%). Twelve percent of program completers reported symptoms of depression or anxiety, and 21% of program completers had non-obesity related comorbidities (asthma [3/92], solid organ replacement [3/92], migraines [1/92], chromosomal anomalies [1/92], midline defect [1/92], or status post bone marrow transplant [1/92]). The quantity of obesity or non-obesity comorbidities at baseline was analyzed as a predictor of improvement in BMI status; having no comorbidities at baseline was associated with an decrease in zBMI, modified BMIz and %BMI_p95_ at the 6^th^ visit (p < 0.05). The presence of obesity, T2D or psychiatric disorder in one parent of the participating youth at baseline was not found to be a significant predictor of BMI reduction (p = 0.20, p = 0.70, p = 0.30).

## Discussion

This study evaluated the effect of a clinic-based, family-centered, multi-disciplinary weight management program on improvement in three BMI metrics (zBMI, modified BMIz and %BMI_p95_). In this study, youth who completed at least six EMPOWER visits experienced modest yet significant improvement in their BMI status across all three metrics. Intriguingly, the reduction in zBMI and modified BMIz was found to be statistically significant while the reduction in %BMI_p95_ was not for the whole group. However, sub-analysis of youth 14–18 years of age, showed a significant reduction across all three BMI metrics, adding to previous literature suggesting that %BMI_p95_ may be a more appropriate metric to evaluate treatment effectiveness for adolescents [25]. This study was not designed to evaluate the utility of various BMI metrics in youth with obesity and severe obesity, but it is essential that the metrics continue to be explored on a population level to determine the best methods to assess intervention effects on weight outcomes in pediatric populations of various ages.

The level of obesity at baseline was found to be the greatest predictor of BMI status improvement, across all three BMI status metrics, even when controlling for other patient characteristics. The present findings are consistent with previous research showing that youth with lower BMI status at baseline show better response to behavioral intervention models. These findings reinforce the need to intervene earlier, before the obesity is severe. Interestingly, unlike previous research, the current investigation found age at treatment initiation did not moderate change in BMI status over the course of treatment when controlling for baseline BMI status. Evaluation of the severity of obesity by age revealed that for our population, those youth with younger age at treatment initiation had higher baseline BMI status, suggesting that for those youth referred to the EMPOWER program, severity of obesity at baseline appears to be more important than age in predicting BMI status improvement. Unlike age, the absence of any comorbid condition at baseline did predict improvement in BMI status [23,29,30]. Therefore the recommendation should not only emphasize early age at first referral, but also suggests that referral should take place prior to the development of additional obesity related comorbidities, rather than utilizing co-morbidities as inclusion criteria for referral to multi-disciplinary weight management treatment.

The predictive value of gender was correlated with severity of obesity at baseline. Males showed a greater reduction in BMI status over the course of treatment; however, female gender was more predictive of BMI status reduction, in our cohort, as the females had lower BMI status at baseline. These findings suggest that the level of obesity at baseline was the main component predicting BMI status reduction over the treatment course, which could explain why in previous work, the predictive effect of gender has not always been consistent between different cohorts studied. Furthermore, in our sample, ethnicity and insurance status did not moderate BMI status change over the treatment course. Given that the majority of our participants were non-Caucasian; this is of particular importance, as obesity is known by most severely affect minority and low-income populations [16]. These findings suggest that the individualized, multidisciplinary approach successfully adapts to each family’s needs and results in equal effectiveness across various types of patient groups.

### Limitations

There are several limitations that should be considered when interpreting our results. Participant data was retrospectively collected from the electronic health record, documented in the clinical setting, rather than prospectively in a research environment, resulting in potentially diminished data quality. In addition, the results reflected changes over an initial 6–9 month treatment period, so the long-term effects of the EMPOWER program on weight maintenance are not known. Finally, although BMI status is a commonly used metric to evaluate these clinical interventions, other psychological and metabolic parameters including indicators of cardio-metabolic health improvement, i.e. increases in physical activity, improvement in eating behaviors, and increased quality of life, must be further investigated to better understand the intervention’s health effects. Finally, a major limitation is the lack of untreated randomized age-matched control groups. However, in current literature of pediatric obesity interventions and the natural trajectory for youth with obesity, it is well described that weight and BMI of youth not receiving any intervention continues to increase [21].

## Conclusions

The only consensus guidelines available for the treatment of obesity in pediatrics recommend clinic- based, family-centered, multi-disciplinary weight management programs. Participation in a clinical program grounded in these recommendations, lead to modest improvements in BMI status in treatment seeking youth. However, the implementation of these programs is resource intensive and only results in modest BMI status improvement. Therefore, further evaluation is required to not only better understand how youth- specific characteristics affect change in BMI trajectory when in treatment, thereby improving patient selection and to optimization of utilization of health care resources, but also to develop innovative behavioral interventions with improved effectiveness in this high risk population.

## Abbreviations

(modified BMIz): Modified BMI Z-score
(zBMI): Body Mass Index Z-score
(%BMI_p95_): Percent over the 95^th^ percentile
